# Significant Changes in the Levels of Secreted Cytokines in Brains of Experimental Antiphospholipid Syndrome Mice

**DOI:** 10.1155/2012/404815

**Published:** 2012-02-22

**Authors:** Assaf Menachem, Joab Chapman, Aviva Katzav

**Affiliations:** ^1^Department of Physiology and Pharmacology, Sackler Faculty of Medicine, Tel Aviv University, 69978 Tel Aviv, Israel; ^2^Department of Neurology and Joseph Sagol Neuroscience Center, Sheba Medical Center, 52621 Ramat Gan, Israel; ^3^Sackler Faculty of Medicine, Tel Aviv University, 69978 Tel Aviv, Israel

## Abstract

Antiphospholipid syndrome (APS) is characterized by thromboses and neuropsychiatric manifestations possibly linked to brain inflammation. In order to examine the levels of proinflammatory and anti-inflammatory cytokines in experimental APS (eAPS) mice brains, we measured the levels of TNF-**α**, IFN-**γ**, and IL-10 in brain homogenates (cytosolic fractions) and in brain slices (secreted level) at 6, 15, and 24 weeks after immunization. We induced eAPS by immunization of Balb/c mice with **β**_2_-glycoprotein I (**β**_2_GPI), the major autoantigen in the disease and controls with adjuvant alone. We found increased levels of secreted TNF-**α** in eAPS mice for the entire experiment period. Cytosolic and secreted IL-10 and IFN-**γ** levels in eAPS mice were lower at 6 and 15 weeks and higher at 24 weeks after immunization. The results suggest that brain disease in APS is associated with significant and complex changes in proinflammatory and anti-inflammatory cytokines.

## 1. Introduction

The antiphospholipid (Hughes) syndrome (APS) is an autoimmune disorder, manifested by thromboembolic events (arterial and venous), recurrent spontaneous abortions, thrombocytopenia, and elevated titers of circulating antiphospholipid antibodies (aPL) [1]. Most aPL are autoantibodies directed against a complex of phospholipids and *β*_2_-glycoprotein I (*β*_2_GPI), a major cofactor in the binding of aPL, causing hypercoagulation and a proinflammatory state [2]. Many neurological manifestations have been described in the APS, but only stroke is well established and accepted as a diagnostic criterion in the disease [3]. Other neurological complications, which still need to be fully established, include seizures, ocular disturbances, dementia, migraine, transverse myelitis, and chorea [4].

A model of experimental APS (eAPS) in mice is induced by immunization with *β*_2_GPI [5]. Previous studies in our lab found behavioral changes in eAPS mice, which include hyperactivity/exploratory behavior and cognitive deficits [6–8]. The CNS manifestation developed over a period of 4-5 months after immunization [9]. The role of aPL in the pathogenesis of APS still needs to be elucidated. It is possible that other immune mediators, apart from autoantibodies, take part in the inflammatory and degenerative processes in the APS brain.

Although APS is considered a B-cell mediated disease [10], T cells also have an important role in the induction and regulation of the disease, by secreting cytokines which modulate the immune response [11]. Cytokines can be divided into two subgroups: proinflammatory cytokines and anti-inflammatory cytokines. The course of many diseases, including several autoimmune diseases, depends upon the balance between these two subgroups [12]. Pro-inflammatory cytokines and chemokines have been reported to have an important role in the development of the clinical manifestation of APS [11, 13]. Previous clinical reports have shown increased serum levels of IL-4 and IL-6 in patients with APS [14, 15]. Another central inflammatory cytokine associated with APS is tissue necrosis factor-*α* (TNF-*α*), levels of which are known to be elevated and reflect pathological processes within the endothelial cells [15, 16].

The cytosolic cytokine content of brain homogenate consists of two parts, one of which is secreted from the activated immune cells or the activated endothelial cells and participates in the modulation of the immune response. The second cytokine fraction is stored within immune and endothelial cells as a reserve and has less immediate effect on the immune response. We hypothesized that the secreted fraction is the one which contributes to development of APS pathogenesis. Our aim in the present study was to compare a number of brain inflammatory cytokines (TNF-*α*, IFN-*γ*, and IL-10) measured both as cytosolic levels (i.e., their concentration in a cytosolic fraction) and as secreted levels (i.e., their concentration when they secretes from brain slices). Changes over the course of 6 months were monitored.

## 2. Materials and Methods

### 2.1. Mice

Female Balb/C mice, aged 8 weeks, were obtained from Harlan Laboratories Limited, Israel. The mice were raised at the Sackler Medical School, Tel Aviv University, animal facility, under standard conditions, 23 ± 1°C, 12 h light cycle (7 am ± 7 pm) with ad libitum access to food and drink. The Tel Aviv University Animal Welfare Committee approved all procedures (M-08-053).

### 2.2. Induction of Experimental APS

 The eAPS group (*n* = 15) was immunized once subcutaneously with 10 *μ*g *β*_2_GPI emulsified in complete Freund's adjuvant (CFA). The control group (*n* = 15) was immunized similarly with CFA alone. In order to monitor changes over time, mice were divided into three groups; each group contained 10 mice, 5 from the eAPS group and 5 from the control group. The first group was sacrificed 6 weeks after immunization, the second group was sacrificed 15 weeks after immunization, and the third group was sacrificed 24 weeks after immunization.

### 2.3. Serology Evaluation

Mice were bled by left ventricle puncture before their brains were perfused and harvested. The sera were separated by centrifugation (9600× g for 10 minutes) and stored at –20°C until assayed. The sera were tested by ELISA for the presence of antibodies to cardiolipin (*β*_2_GPI-dependent) as previously described [10].

### 2.4. Tissue Preparation

 Each mouse, under equithesin anesthesia, was perfused through the heart with PBS (pH 7.4) containing 5 U/mL heparin. After perfusion, the brain was quickly removed and was cut into two halves. One half of the brain was homogenized using disposable rotor homogenizer (OMNI-INC) in 10 volumes of radioimmunoprecipitation assay** (**RIPA) buffer (50 mM Tris-HCl, 150 mM NaCl, pH 7.4) supplemented with 1 mM ethylenediaminetetraacetic acid (EDTA), 1 mM phenylmethylsulphonyl fluoride (PMSF), and a protease inhibitor cocktail (Sigma-Aldrich) and was separated to subcellular fractions detailed below; the second half of the brain was stored at −70°C until it was cut serially into 50 *μ*m thick, coronal sections on a cryostat (Leica). The sections were kept in a cryoprotectant (28% Glycerol, 29% Ethylene glycol in 0.1 M PO_4_) at –20°C until assayed.

### 2.5. Subcellular Fractionation

 All steps were carried at 4°C. The homogenate was centrifuged at 2900 ×g for 20 min. The pellet (nuclear fraction) was resuspended with RIPA buffer containing 1 mM EDTA and protease inhibitor cocktail, whilst the supernatant was centrifuged at 29000 ×g for 45 min. The resulting supernatant (cytosol fraction) was collected and kept at –20°C until assayed.

### 2.6. Determination of Cytokine Levels

 Tumor necrosis factor-alpha (TNF-*α*), interleukin-10 (IL-10), and interferon-gamma (IFN-*γ*) concentrations were measured by specific quantitative sandwich ELISA kits (PeproTech Inc.) according to the manufacturer's instructions. The cytosolic cytokine level was measured in the cytosolic fraction of a whole brain homogenate. In order to measure secreted fraction from the cells, brain slices were incubated for 2 hours at 37°C in PBS (pH 7.4) buffer. The supernatant was collected; its protein concentration was measured by the bicinchoninic acid (BCA) method, and the concentrations of the cytokines were measured by the ELISA kits as for the cytosolic fractions.

### 2.7. Statistical Analysis

 Results are expressed as mean values ± standard error of mean (SEM). Cytokine levels were compared for immunization and time effects between the appropriate groups by means of a univariate 2-way ANOVA. Interaction for group × time was also performed. Post hoc analysis was performed by* t*-test. All analyses were performed by SPSS (Chicago IL). In order to standardize and enable comparison between various experiments, mean of controls were calculated as 100%.

## 3. Results

### 3.1. Autoantibody Levels

**Figure 1:**
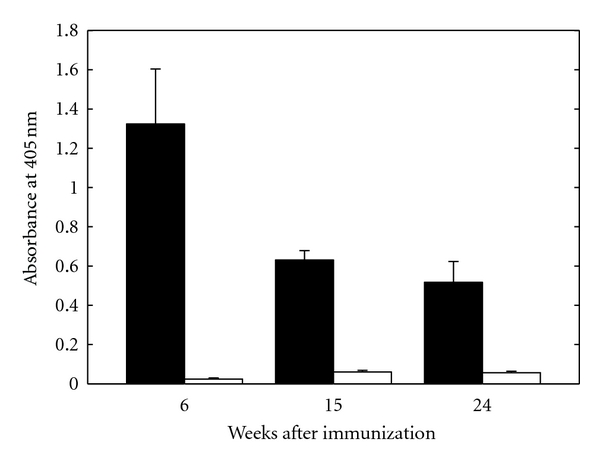
Antibodies to cardiolipin (*β*_2_GPI-dependent) in eAPS (black bars) and adjuvant control mice (white bars) sera 6, 15, and 24 weeks after immunization. Results are presented as the mean absorbance values ± SEM.

Antibody levels to cardiolipin (*β*_2_GPI-dependent) in the sera are presented in Figure 1. Six weeks after immunization eAPS mice developed elevated titers of antibodies to cardiolipin. Antibody levels were significantly higher in eAPS compared to control mice immunized with CFA alone (1.32 ± 0.28 and 0.02 ± 0.01 OD units, resp.,  *P* < 0.001 by *t*-test). Fifteen weeks after immunization, antibody levels in eAPS mice were decreased but still remained significantly higher compared to controls (0.63 ± 0.04 and 0.06 ± 0.01 OD units, resp., *P* < 0.001 by *t*-test). Twenty-four weeks after immunization, antibody levels in eAPS mice were remained significantly higher compared to controls (0.52 ± 0.11 and 0.06 ± 0.01 OD units, resp., *P* < 0.005 by *t*-test).

### 3.2. Secreted versus Cytosolic Cytokine Levels

#### 3.2.1. TNF-*α* Level

**Figure 2:**
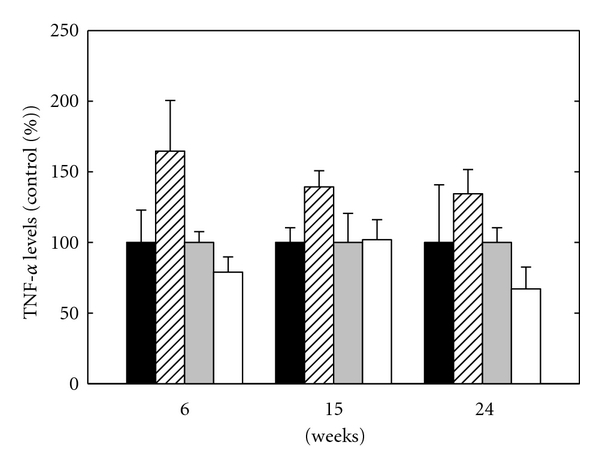
Secreted TNF-*α* levels of adjuvant mice (black bars) and eAPS mice (upward diagonal bars) versus cytosolic TNF-*α* levels of adjuvant mice (grey bars) and eAPS mice (white bars). The average of TNF-*α* level of the adjuvant control group was defined as 100%, and the results expressed as percentage of control group at the same time point. Results are presented as the mean ± SEM.

 Cytosolic and secreted TNF-*α* levels of eAPS and control mice are presented in Figure 2. TNF-*α* levels were calculated as percentage of control group. Secreted TNF-*α* level in the control group remained stable at 6 and 15 weeks (3.7 ± 1.3 pg/mL and 3.7 ± 0.3 pg/mL, resp.) and decreased slightly at 24 weeks (2.1 ± 0.8 pg/mL). In the eAPS group, secreted TNF-*α* level was at its highest level at 6 weeks, while, at 15 weeks, it decreased and remained at the same level at 24 weeks. As can be seen, secreted TNF-*α* levels were higher in the eAPS group compared to the control group at 6, 15, and 24 weeks. Analysis for the effect of group and time by univariate 2-way ANOVA revealed a significant effect of group (*P* = 0.02). There was no significant effect for the interaction group × time (*P* > 0.5) indicating that the behavior of both groups was similar over time. Post hoc analysis by *t*-test revealed significant difference between groups at 6 and 15 weeks (*P* < 0.05).

Cytosolic TNF-*α* level in the control group was stable at 6 and 15 weeks (4.2 ± 0.7 *μ*g/mL and 4.0 ± 0.8 *μ*g/mL, resp.) and increased at 24 weeks (5.9 ± 1.0 *μ*g/mL). In the eAPS group, cytosolic TNF-*α* level was at its lowest level at 6 weeks, while, at 15 weeks, it was increased and remained at the same level at 24 weeks. As can be seen, cytosolic TNF-*α* levels were lower in the eAPS group compared to the control group at 6 and 24 weeks, while, at 15 weeks, levels in the eAPS group and the control group were similar. Analysis for the effects of group and time by univariate 2-way ANOVA revealed a nonsignificant effect of group and time (*P* < 0.1, *P* = 0.17 resp.). There was no effect for the interaction group × time (*P* > 0.3).

#### 3.2.2. IL-10 Level

**Figure 3:**
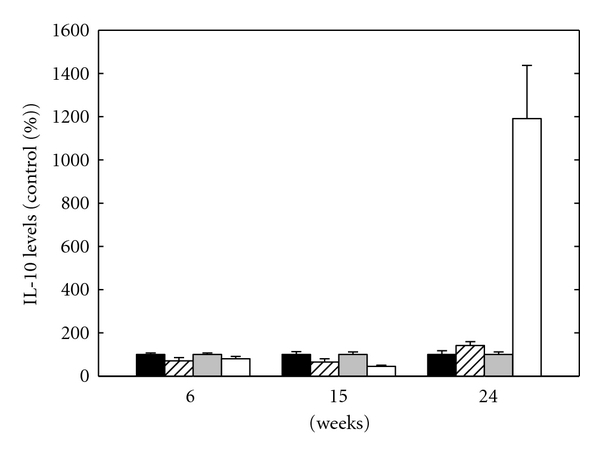
Secreted IL-10 levels of adjuvant mice (black bars) and eAPS mice (upward diagonal bars) versus cytosolic IL-10 levels of adjuvant mice (grey bars) and eAPS mice (white bars). The average of IL-10 level of the adjuvant control group was defined as 100%, and the results expressed as percentage of control group at the same time point. Results are presented as the mean ± SEM.

Cytosolic and secreted IL-10 levels of eAPS and control mice are presented in Figure 3. IL-10 levels were calculated as percentage of control group. Secreted IL-10 level in the control group was at its highest level at 6 weeks (25.7 ± 1.4 pg/mL) and gradually decreased at 15 and 24 weeks (15.7 ± 2.1 pg/mL and 9.9 ± 1.7 pg/mL, resp.). In the eAPS group, secreted IL-10 was at its lowest level at 15 weeks; at 6 and 24 weeks, the levels were similar. As can be seen, secreted IL-10 level in the eAPS group compared to the control group was lower at 6 and 15 weeks and higher at 24 weeks. Analysis for the effect of group and time by univariate 2-way ANOVA revealed a significant effect of time (*P* < 0.03). There was a significant effect for the interaction of group × time (*P* < 0.02) due to the decrease over time in the control group level.

Cytosolic IL-10 level in the control group was 7.6 ± 0.5 *μ*g/mL, 11.5 ± 1.4 *μ*g/mL, and 84.0 ± 17.4 *μ*g/mL, at 6, 15, and 24 weeks, respectively. In the eAPS group, cytosolic IL-10 level decreased to its lowest level at 15 weeks; at 24 weeks, IL-10 increased to its highest level. Similarly to the secreted IL-10 level, cytosolic IL-10 level in the eAPS group compared to the control group was lower at 6 and 15 weeks and higher at 24 weeks. Analysis for the effect of group and time by univariate 2-way ANOVA revealed a significant effect of group (*P* < 0.01) and time (*P* < 0.01). There was a significant effect for the interaction of group × time (*P* < 0.01). Post hoc analysis by *t*-test revealed a significant effect at 15 and 24 weeks (*P* < 0.05) and a trend toward increased levels of cytosolic IL-10 in the control group at 6 weeks (*P* = 0.16).

#### 3.2.3. IFN-*γ* Level

**Figure 4:**
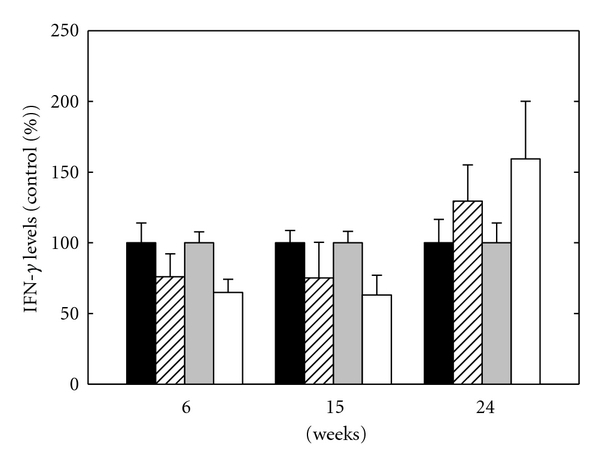
Secreted IFN-*γ* levels of adjuvant mice (black bars) and eAPS mice (upward diagonal bars) versus cytosolic IFN-*γ* levels of adjuvant mice (grey bars) and eAPS mice (white bars). The average of IFN-*γ* level of the adjuvant control group was defined as 100%, and the results expressed as percentage of control group at the same time point. Results are presented as the mean ± SEM.

 Cytosolic and secreted IFN-*γ* levels of eAPS and control mice are presented in Figure 4. IFN-*γ* levels were calculated as percentage of control group. Secreted IFN-*γ* level in the control group was at its highest level at 6 weeks (9.8 ± 2.1 pg/mL), and, at 15 and 24 weeks, the levels gradually decreased (8.8 ± 0.9 pg/mL and 5.0 ± 0.8 pg/mL, resp.). In the eAPS group, secreted IFN-*γ* level was stable for the whole period. As can be seen, secreted IFN-*γ* level in the eAPS group compared to the control group was lower at 6 and 15 weeks and higher at 24 weeks. Analysis for the effect of group and time by univariate 2-way ANOVA revealed no significant effect for either group or time (*P* = 0.19 for both). There was no effect for the interaction of group × time (*P* > 0.5).

Cytosolic IFN-*γ* level in the control group was 6.3 ± 0.5 *μ*g/mL, 7.5 ± 0.6 *μ*g/mL, and 4.5 ± 0.6*μ*g/mL at 6, 15, and 24 weeks, respectively. In the eAPS group, cytosolic IFN-*γ* level was stable at 6 and 15 weeks and increased at 24 weeks. As can be seen, cytosolic IFN-*γ* level in the eAPS group compared to the control group was lower at 6 and 15 weeks and higher at 24 weeks. Analysis for the effect of group and time by univariate 2-way ANOVA revealed no significant effect for either group or time (*P* > 0.3 or *P* > 0.5, resp.). There was a significant effect for the interaction of group × time (*P* < 0.05).

## 4. Discussion

In the present study, we measured inflammatory secreted and cytosolic cytokines levels in eAPS and adjuvant mice brains during a 24-week period. Secreted TNF-*α* level was higher in eAPS mice compared to adjuvant mice for the whole period. Higher secreted TNF-*α* levels in eAPS mice are in line with previous studies that found higher TNF-*α* levels in eAPS mice and APS patients [15, 16]. On the other hand, cytosolic TNF-*α* levels were somewhat lower in eAPS mice compared to adjuvant mice at 6 and 24 weeks and similar at 15 weeks after immunization.

Explanation for the lower cytosolic TNF-*α* level in eAPS mice at 6 weeks after immunization might be a high secretion of TNF-*α* during the pathological processes, resulting in emptying the cell reserves. As mentioned above, the cytosolic cytokine content of brain homogenate consists of a stored fraction and a secreted fraction. The first fraction is stored within immune and endothelial cells as a reserve and has less immediate effect on the immune response. The second fraction is secreted from the activated immune cells or the activated endothelial cells and participates in the modulation the immune response. We hypothesize the levels of secreted cytokines reflect, as closely as possible, the condition of cytokines levels *in vivo*, since the tissue and the cells structures remain unbroken. We have previously measured TNF-*α* level in mice whole brain homogenate. TNF-*α* level was found to be significantly higher in eAPS mice compared to adjuvant mice [16]. We hypothesize that the TNF-*α* level measured in mice whole brain homogenate is similar to the secreted TNF-*α* level rather than to the cytosolic TNF-*α* level measured in mice cytosolic brain fraction. In comparison to the clean cytosolic fraction, whole brain homogenate contains other components which may mask the cytokine fraction stored within the immune and endothelial cells.

Other important cytokines in the development of the inflammatory process are IL-10 and IFN-*γ*. Cytosolic and secreted IL-10 and IFN-*γ* levels in eAPS mice were lower at 6 and 15 weeks and higher at 24 weeks after immunization compared to adjuvant mice. The low stable secreted and cytosolic IFN-*γ* levels in the eAPS group is in agreement with previous study which showed a rise in IFN-*γ* in eAPS mice after treatment with anti-idiotypic monoclonal antibody (MoAb) [17]. IFN-*γ* is a proinflammatory cytokine and has a major role in the inflammatory process. Low secreted and cytosolic IFN-*γ* levels in eAPS mice might be a result of upegulation of other cytokines which inhibit its production. Proinflammatory cytokines have an important role in regulation of haemostatic balance in both physiological and pathologic states. TNF-*α* influences endothelial cells function by upregulating tissue factor (TF) levels [18, 19]. High levels of TF trigger endothelial cells to change their antithrombotic properties into procoagulant state. Beside its ability to induce procoagulant activity, TNF-*α* also inhibits the thrombomodulin/protein C anticoagulation pathway and affects fibrinolysis by upregulating both urokinase-type plasminogen activator and plasminogen activator inhibitor-1 (PAI-1) [20].

While TNF-*α* contributes to the inflammatory process, IL-10 has an important role in modulating the inflammatory response and autoimmune disease. Impairment of the balance between the inflammatory process and the anti-inflammatory response may lead to disproportionate pathology or immunosuppression. Initially, IL-10 was considered as typical Th2 cytokine. It was identified as a product of activated Th2 cells. Recent studies have shown that IL-10 is also produced by Th1, Th0, regulatory T (Tr1) cells, and in mice also by activated macrophages and B cells [21]. IL-10 downregulates the inflammatory response by blocking the production of a number of cytokines, including IL-2, IFN-*γ*, and TNF-*α* [22]. Therefore, reduced IL-10 levels in eAPS mice may down modulate the immunosuppressive effects, resulting in a proinflammatory process. In addition, low IL-10 levels enable TNF-*α* unregulated production, resulting in procoagulant state. Moreover, during the B-cell activation, IL-10 delivers negative signals that promote the apoptosis of B cells [23]. Decreased IL-10 levels can be associated with lymphocyte activation, which leads to the continuation of the autoimmune response. At 24 weeks, both total and secreted IL-10 levels in eAPS mice increased. This is compatible with an anti-inflammatory stage of disease occurring in conjunction with the drop in the levels of antibodies and TNF-*α*. Explanation for the increased IL-10 levels might be their positive stimulation on B cells. IL-10 has a biphasic effect on B cells. On activated B cells, IL-10 promotes both their proliferation and differentiation into antibody secreting cells.

The importance of immune mediators in APS was demonstrated in previous studies. Several studies in murine model of APS showed that in pregnancy loss, some of the damage is caused by aPL-induced complement activation [24, 25]. Heparin, an anticoagulant has long been known to inhibit complement activity [26, 27]. Girardi et al. found that heparin, but neither fondaparinux nor hirudin, inhibited the generation of complement split products and protected mice from fetal loss caused by aPL antibodies [28]. Some CNS manifestations of APS are caused by inflammation, cytokines or antibody-mediated tissue damage, and, therefore, antithrombotic therapy in APS is not sufficient.

The results suggest that course of the inflammatory process in eAPS depends upon the balance between proinflammatory and anti-inflammatory cytokines. Today, the accepted treatment for APS is antithrombotic therapy. Our results in line with previous cytokine studies may indicate a new therapeutic approach to APS. Drugs against proinflammatory cytokines may rebalance the cytokines levels and moderate the inflammatory response.
